# Antithrombotic Therapy after Percutaneous Left Atrial Appendage Closure: Evidence, Challenges and Future Directions

**DOI:** 10.31083/j.rcm2412343

**Published:** 2023-12-12

**Authors:** Roberto Galea, Lorenz Räber

**Affiliations:** ^1^Department of Cardiology, Inselspital, Bern University Hospital, University of Bern, 3010 Bern, Switzerland

**Keywords:** left atrial appendage closure, antithrombotic therapy, drug regimen, device related thrombus

## Abstract

Percutaneous left atrial appendage closure (LAAC) has been established in clinical practice as an attractive alternative to oral anticoagulation for preventing stroke in patients with atrial fibrillation and high bleeding risk. The devices approved in Europe and United States (US) for percutaneous LAAC contain metal and antithrombotic therapy is strongly recommended after their implantation to prevent apposition of thrombus on the atrial surface of the device during endothelialization. However, there is still uncertainty regarding the optimal antithrombotic drug regimen following device implantation in view of the incomplete understanding of the LAAC device healing process, the lack of randomized clinical trials comparing different antithrombotic agents after LAAC and the heterogeneous bleeding risk of patients undergoing LAAC. Thus, this review aims to evaluate the available evidence and the remaining challenges related to the post-LAAC antithrombotic regimens. Furthermore, common clinical scenarios associated with challenging management of antithrombotic therapy after LAAC and potential future directions, will be discussed.

## 1. Introduction

Atrial fibrillation (AF) increases the risk of stroke by 5-fold [1, 2] and oral anticoagulant (OAC) therapy represents the first-line therapy for its prevention. However, OAC carries several limitations such as the associated bleeding risk, limited efficacy and potential issues with compliance, which has led to the search for alternative approaches.

The majority of all cardiac thrombi in patients with AF originate from the left atrial appendage (LAA) [3]. Thus, the percutaneous LAA closure (LAAC) procedure, consisting of the exclusion of the LAA cavity from the circulation by implanting a cardiovascular device at LAA ostium, has been established in clinical practice as an attractive alternative to OAC for preventing stroke in AF patients, especially in those with high bleeding risk. Watchman (Boston Scientific, Natick, MA, USA) and Amulet (St. Jude Medical/Abbott, Nathan Lane North Plymouth, MN, USA) are the two most frequently implanted LAAC devices worldwide [4]. Watchman FLX is a self-expanding nitinol cage covered with a porous polyethylene terephthalate membrane on the proximal face, secured with fixation barbs located circumferentially, whereas Amulet consists of a distal hook-crowned lobe for anchoring in the lumen of the LAA and a proximal disc for excluding the LAA ostium. Since the percutaneous LAAC devices so far are at least partially metallic, antithrombotic therapy is strongly recommended following implantation to prevent device related thrombus (DRT) during the endothelialization process, comparable to the situation following coronary stent implantation. However, there is still uncertainty regarding the optimal regimen following percutaneous LAAC in view of the incomplete understanding of the LAAC device healing process, lack of standardized definition of device neo-endothelialization [5], uncertainty of the impact of peri-device leaks (PDL) on the risk of recurrent thromboembolic events, lack of studies comparing different antithrombotic agents after LAAC and the heterogeneity of patients undergoing LAAC in terms of bleeding risk. Thus, this review evaluates the available evidence, the remaining challenges and potential future directions related to the post-LAAC antithrombotic regimens.

## 2. Antithrombotic Therapy Regimens after LAAC

Few animal studies have shown that a complete endothelialization of the atrial surface of LAAC devices is not completed prior to 90 days [6, 7]. In humans, this process may be longer as compared with dogs [8]. Thus, the post-LAAC antithrombotic therapy regimen recommended in the context of the randomized controlled trials (RCT) leading to the US Food and Drug Administration (FDA) approval of Watchman [9, 10] and Amulet [11], consisted of the use of two antithrombotic agents for at least 6 months. With the primary endpoint completion of the above trials and the consequent market release of the two devices, the FDA recommended the following drug regimens: in Watchman patients, OAC plus Aspirin for 45 days should be given, followed by 4.5 months of dual antiplatelet therapy (DAPT) and then by Aspirin alone; on the other hand, Amulet implantation should be followed by DAPT or OAC plus Aspirin for 45 days, followed by DAPT for 4.5 months and then by Aspirin alone (Table 1). However, nowadays the majority of patients undergoing LAAC in clinical practice (especially outside of the US) are elderly, have high bleeding risk and are deemed non-eligible even for short-term OAC or 6 months DAPT [12, 13]. It is therefore not surprising that antithrombotic regimens in clinical practice widely differ from authority recommendations.

**Table 1.** **Devices for percutaneous left atrial appendage closure approved in Europe and United States and the recommended antithrombotic therapy**.

**LAAC device name**	**FDA approval**	**Antithrombotic therapy recommended by FDA after LAAC**	**CE mark**	**Antithrombotic therapy recommended by EMEA after LAAC**	Main prospective studies*			**Patients (No)**
					**Study design**	**Study arms**	**Study name**	
Watchman 2.5	2015	OAC+ASA for 45 days followed by 4.5 months of DAPT; then ASA alone	2005	OAC+ASA or DAPT for 45 days followed by 45 days (at least) of DAPT; then ASA alone (for at least 12 months)	RCT	Watchman 2.5 vs. VKA	Protect AF	463
					RCT	Watchman 2.5 vs. VKA	Prevail	269
					RCT	Watchman 2.5 vs. Amulet	Amulet IDE	944
					MCOS	Watchman 2.5 Registry	Ewolution	1021
Watchman FLX	2020		2019		MCOS	Watchman FLX Registry	Pinnacle FLX	400
Amulet	2021	DAPT or OAC+ASA for 45 days followed by DAPT for 4.5 months, followed by ASA alone	2013	DAPT of variable duration followed by ASA alone (for at least 6 months)	RCT	Amulet vs. Watchman 2.5	Amulet IDE	934
					MCOS	Amulet Registry	Amulet global registry	1088

*The studies include all the dedicated randomized clinical trials and the largest available observational studies. FDA, food and drug administration; LAAC, left atrial appendage closure; CE, conformity european; EMEA, european medicines evaluation agency, OAC, oral anticoagulation; ASA, aspirin; DAPT, dual antiplatelet therapy; RCT, randomized clinical trial; MCOS, multicenter observation study; FLX, Watchman FLX; VKA, Vitamin K Antagonists; AF, atrial fibrillation; IDE, investigational device exemption.

The largest prospective multicenter observational studies conducted outside of the US reportedly showed that shorter DAPT durations may be safe and efficacious following implantation of Watchman [14] or amplatzer cardiac plug (ACP)/Amulet [15, 16] devices. As a consequence, the Instructions for Use (IFU) of Watchman and Amulet devices labeled with CE mark, allowed prescription after LAAC of variable duration DAPT followed by Aspirin alone (Table 1).

The different drug regimens recommended after LAAC between American and European competent authorities, the lack of RCTs comparing various antithrombotic post-LAAC regimens and the heterogeneous risk class of patients commonly submitted to LAAC, led to a diversification of the post-LAAC antithrombotic regimens in clinical practice that can be summarized in five potential strategies (Table 2, Ref. [17, 18, 19, 20, 21, 22, 23]):

∙ Combination of OAC and antiplatelet therapy,

∙ OAC alone,

∙ DAPT,

∙ Single Antiplatelet Therapy (SAPT),

∙ No antithrombotic therapy.

Evidence related to the efficacy and safety of each drug regimen will be discussed below.

**Table 2.** LAAC studies and their antithrombotic regimen within 45 days of procedure*.

**Main anti-thrombotic regimen**	**Study name**		**Study design**	**Device**	**Patient (No)**	**Age (mean)**	**CHA2DS2VASC score (mean)**	**HASBLED score (mean)**	**History of MB (%)**	**Clinical- FU time (months)**	**Ischemic stroke (events/patient-yrs)**	**Major bleeding (events/patient-yrs)**	**Imaging-FU time (months/method)**	**DRT (%)**
VKA+SAPT	Protect AF		RCT	W2.5	463	71.7	3.4	1–2 **	13.1	48	1.4	3.1 µ	1.5/TEE	3.4
	Prevail		RCT	W2.5	269	74	4.0	1–2 **		48	1.7		NA	NA
	Amulet IDE		RCT	W2.5	944	75.1	4.7	3.3	26.5	18	1.8	10	1.5/TEE	4.5
DOAC+SAPT	Pinnacle FLX		MCOS	FLX	400	73.8	4.2	2.0	NA	12	2.6	7.9	1.5/TEE	0.2
	Della Rocca *et al*. 2021 [17]		MCOS	W2.5	198	74.8	4	3	53.5	13	1.1	2.3 ¥	1.5/TEE	2.1
OAC	VKA	Fu G. *et al*. 2022 [22]	SCOS	W2.5	77	69.8	4.5	3.1	23.4	NA	NA	NA	1.5/TEE-CCTA	4.2
	DOAC				291	69.6	4.6	3.0	17.6					0.7
	VKA	Enomoto Y. 2017 [23]	MCOS	W2.5	212	75	4.1	2.7	NA	NA	NA	NA	1.5-4/TEE-CCTA	0.5
	DOAC				214	76	3.8	2.4						0.9
DAPT	Ewolution		MCOS	W2.5	1021	73.4	4.5	2.3	31.3	24	1.3	2.7	1.5/TEE	4.1
	Amulet Registry		MCOS	Amulet	1078	72.5	4.2	3.3	72	24	2.2	7.2	1.5/TEE	1.6
	Amulet IDE		RCT	Amulet	934	75	4.5	3.2	29	18	1.7	10.6	1.5/TEE	3.3
	Patti *et al*. 2020 [21]		MCOS	Amulet/W2.5	330	74.9	3.9	3.3	55	12	2.1	6.7	2/TEE	0.9
SAPT					280	76	4.3	3.4	56		1.8	2.9		0.7
	Nielsen-Kudsk *et al*. 2017 [18]		MCOS	ACP/Amulet	151	71.9	3.9	4.2	100	6	1.7	3.5	NA	NA
	Korsholm *et al*. 2017 [19]		SCOS	Amulet	107	73.2	4.4	4.1	82.2	24	4.7	5.6	1.5/TEE-CCTA	1.9
	Pouru *et al*. 2020 [20]		SCOS	Amulet	81	74.5	4.5	3.1	NA	35	1.7	1.6 ¥	NA	NA

*For each antithrombotic regimen, studies (n = 3 whenever possible) with the largest population size have been reported. **HASBLED was 1 or 2 in roughly 70% of patients randomized to LAAC. Defined according to the Bleeding Academic Research Consortium (BARC) as BARC ≥3. It included any overt bleeding with either a decrease in hemoglobin of ≥3.0 g/dL, transfusion of ≥1 unit of packed red cells, requiring intervention, bleeding at a critical site (intracranial, intraspinal, intraocular, pericardial, intramuscular with compartment syndrome, or retroperitoneal), or fatal bleeding. µ It included pericardial effusion requiring drainage, intracranial bleeding, or GI bleeding requiring transfusion. ¥ According to the International Society on Thrombosis and Hemostasis (ISTH) criteria. It included either a decrease in hemoglobin of ≥2.0 g/dL during a 24-h period, transfusion of ≥2 units of packed red cells, bleeding at a critical site (intracranial, intraspinal, intraocular, pericardial, intramuscular with compartment syndrome, or retroperitoneal), or fatal bleeding. OAC, oral anticoagulation; DAPT, dual antiplatelet therapy; SAPT, single antiplatelet therapy; VKA, vitamin K antagonist; DOAC, direct oral anticoagulant; W2.5, watchman; FLX, watchman FLX; ACP, amplatzer cardiac plug; RCT, randomized clinical trial; MCOS, multi-center observation study; SCOS, single-center observation study; MB, major bleeding; FU, follow-up; SE, systemic embolism; DRT, device related thrombus; NA, not available; TEE, tranesophageal echocardiography; CCTA, cardiac computed tomography angiography; LAAC, left atrial appendage closure; AF, atrial fibrillation; IDE, investigational device exemption.

### 2.1 Combination of Anticoagulation and Antiplatelet Therapy

The post-LAAC drug regimen recommended in the context of the RCTs leading to FDA approval of Watchman and Amulet devices included the combination of OAC (in particular Vitamin K Antagonists [VKA]) and Aspirin.

The PROTECT AF trial was a multicenter RCT of 707 non-valvular AF patients deemed eligible for OAC, which was designed to test whether LAAC with Watchman was non-inferior to VKA for a composite of stroke, systemic embolism, or cardiovascular death [10]. After a mean follow-up of 4 years, the composite primary endpoint was significantly lower in LAAC as compared to VKA (8.4% vs. 13.9%; rate ratio [RR]: 0.60; 95% credible interval [CrI]: 0.41–1.05), meeting the pre-specified criterion for non-inferiority. Furthermore, in the LAAC group significantly lower safety events such as hemorrhagic strokes (RR: 0.15; 95% CrI: 0.03–0.49) and all-cause fatal events (hazard ratio [HR]: 0.66; 95% CrI: 0.45–0.98) were observed, although the study was not powered for these [24]. The PREVAIL trial was a confirmatory RCT with a similar design to the PROTECT AF trial, showing improved intraprocedural LAAC safety as compared to the previous trial (rate of severe safety events: 4.5% vs. 8.7% respectively) [9]. The antithrombotic therapy mandated after LAAC in the study protocol of the above two trials, which was subsequently recommended by the FDA for Watchman device, consisted of VKA plus Aspirin for 45 days followed by 4.5 months of DAPT and then Aspirin alone. The 5-year outcomes of both PREVAIL and PROTECT trials were combined in a meta-analysis that showed similar incidence of composite of ischemic outcomes between LAAC and VKA groups (HR: 0.82; 95% Confidence Interval [CI]: 0.58–1.17; *p* = 0.27) but lower rates of mortality (HR: 0.73; 95% CI: 0.54–0.98; *p* = 0.035) and non-procedure-related major bleeding (HR: 0.48; 95% CI: 0.32–0.71; *p* = 0.0003) in the LAAC group [25]. Based on these trials, combining OAC with SAPT for at least 45 days following Watchman implantation appears a safe and efficient strategy for preventing ischemic events and DRT (the Protect AF trial reported a rate of 3.4% at 45-day tranesophageal echocardiography (TEE)), without significantly increasing bleeding events. However, it is important to underline the low risk population enrolled in these trials. The mean age was 72.6 ± 8.4 years (although no age limit was stated) and AF patients not eligible for long-term anticoagulation, or with thrombocytopenia or anemia, or sick patients with a life expectancy shorter than 2 years, were excluded from the two pivotal Watchman trials [9, 10]. Accordingly, the rate of major bleeding in the metanalysis including these two studies was exceedingly low (3.1% at five years) [25]. Based on recent improvements in terms of technical success in recent studies (0.9–2.7% vs. 5–12%) [14, 26], and the introduction of new device iterations such as Watchman FLX (correlated to lower risk of DRT as compared to the previous Watchman 2.5) [27], a less intense antithrombotic therapy regimen following LAAC appears reasonable to mitigate the important bleeding risk following LAAC.

Regarding the Amulet device, the recent Amulet investigational device exemption (IDE) trial, a multicenter RCT comparing Watchman 2.5 vs. Amulet in 1878 patients with AF deemed eligible for short-term OAC therapy, reported non-inferiority of Amulet as compared to Watchman 2.5 for both primary safety endpoint (composite of procedure-related complications, all-cause death, or major bleeding at 12 months: 14.5% vs. 14.7%; *p* < 0.001 for non-inferiority) and primary efficacy endpoint (composite of ischemic stroke or systemic embolism at 18 months: 2.8% vs. 2.8%; *p* < 0.001 for non-inferiority) [28]. In this RCT, the drug regimen recommended after Amulet implantation (which is now recommended by the FDA), consisted of DAPT or OAC plus Aspirin for 45 days followed by DAPT for 4.5 months. However, at hospital discharge, only one-fifth of Amulet patients were on OAC plus Aspirin while the majority (75.7%) were receiving DAPT. Most (82.0%) Watchman patients were discharged on warfarin plus aspirin. At three years after LAAC, no difference in terms of thromboembolic events or major bleeding was observed between the two study groups [29]. Of note, in the Watchman group, the annualized major bleeding rate (6.9%) was significantly higher when compared to that reported by the previous Watchman trials (at 5 years the rate of major bleeding was 3.1% patient-yrs) [25]. This apparent difference might be explained by the higher risk population included: mean age 75.0 ± 7.6 vs. 72.6 ± 8.4 years; mean CHA2DS2VASC score 4.6 vs. 3.6; mean HASBLED score: 3.2 vs. 1.9; patients enrolled in Amulet IDE have to be deemed suitable by a multidisciplinary team only for short term OAC (instead of long term as occurred in both Protect-AF and Prevail trials) [28]. Interestingly, among patients enrolled in the Amulet IDE trial, a higher rate of peri-procedural pericardial effusion was observed in patients discharged with OAC versus those without OAC (5.3% vs. 1.8%; *p* = 0.008) [28]. Consistently, in the propensity-matched analysis including 1527 patients enrolled in the main prospective studies with Watchman 2.5, administration of OAC at discharge was associated with an increase of periprocedural bleeding as compared to DAPT [30].

The majority of patients with non–valvular AF receive direct oral anticoagulants (DOAC), which were not approved for use at the time of the two pivotal LAAC RCTs [31]. The combination of DOAC plus aspirin after LAAC for at least 45 days followed by DAPT until 6 months after procedure was recently tested in the PINNACLE FLX trial, a prospective multicenter observational study including AF patients with contraindication to long-term OAC undergoing LAAC with Watchman FLX in the US [26]. This study showed encouraging outcomes in terms of ischemic stroke (2.6% at 1 year), DRT (0.2% at 45-day TEE) and major bleedings rates (7.9% at 1 year), despite a mean age of 74 years, a mean CHA2DS2Vasc Score of 4.3 and a mean HASBLED Score of 2. Collectively, these data suggest that moving to VKA after LAAC in a patient already treated with a DOAC may be unnecessary. Low-dose DOAC is increasingly used as an alternative to VKA in combination with Aspirin after LAAC [32]. Della Rocca *et al*. [17] recently showed in a multi-center cohort of 555 patients undergoing successful LAAC that half-dose DOAC regimen (Aspirin plus half-dose DOAC [apixaban 2.5mg twice a day in almost 90% of cases] for 45 days followed by half-dose DOAC) at 13 months significantly reduced rates of DRT (0.0% vs. 3.4%; *p* = 0.009), non-procedural major bleeding (0.5% vs. 3.9%; *p* = 0.018) and composite of DRT, thromboembolic events and major bleeding events (1.0% vs. 9.5%; *p* = 0.002) as compared to standard antithrombotic therapy (Aspirin plus DOAC for 45 days followed by DAPT for 4.5 months, and then SAPT).

Collectively, short-term OAC (DOAC or VKA) in combination with aspirin for at least 45 days is the most frequently tested antithrombotic regimen after LAAC in RCTs and should therefore be routinely recommended in patients with low bleeding risk. The potential cohort for this drug regimen might include those AF patients submitted to LAAC due to recurrent minor bleedings under OAC, thromboembolic events under OAC, reduced OAC compliance/tolerance or OAC refusal. Half-dose DOAC in association with Aspirin is a promising alternative, however, a dedicated RCT is required prior to recommending this treatment regimen.

### 2.2 Anticoagulation Alone

A potential alternative to the combined antithrombotic therapy described above, is the anticoagulation alone drug regimen. Although this pharmacological strategy has never been tested in RCTs, the rationale supporting the use of this strategy after LAAC includes several observations. Rodés-Cabau *et al*. [33] demonstrated a significant increase of coagulation activation markers seven days after intervention without any changes of platelet activation markers in a single center cohort of forty-three AF patients submitted to successful LAAC. Consistently, Asmarats *et al*. [34] compared the post-Watchman prothrombotic status between thirty patients receiving OAC and forty-eight patients receiving antiplatelet therapy. In the OAC group, not only was the activation of the coagulation system significantly lower as compared to the antiplatelet group, but no DRTs were observed. Of note, all cases of DRT observed in the antiplatelet group had a significantly greater increase in the levels of prothrombotic markers [34]. The pilot ADRIFT study randomized 105 patients submitted to successful LAAC to receive rivaroxaban 10 mg, rivaroxaban 15 mg, or DAPT. Again, not only were reduced doses of rivaroxaban associated with significantly lower thrombin generation when compared with DAPT, but no DRT was observed in both OAC groups at 3-month follow-up (0% vs. 0% vs. 6.1%) [35]. Finally, the complementary effect of antiplatelet and anticoagulant therapy in AF patients not submitted to LAAC was tested by Dentali *et al*. [36] in a systematic review and metanalysis including ten RCTs comparing aspirin plus OAC with OAC therapy alone in patients with at least 3 months of follow-up. The authors observed in more than 4000 patients similar thromboembolic event rates in AF patients receiving combined aspirin-OAC therapy compared with OAC therapy alone. As expected, the rate of major bleeding was higher in patients receiving combined therapy compared with OAC therapy alone [36]. These observations in addition to the progressively increased bleeding risk of patients submitted in clinical practice to LAAC, led to an increase in incidence of discharging patients under OAC alone [22, 23], as occurred in the 5–27% of patients enrolled in the recent large multicenter studies [14, 37, 38, 39]. In this regard, a recent analysis of the American registry including 31,994 AF patients successfully treated with Watchman 2.5 implantation in the two years 2016–2018, showed that the adjusted risk of any adverse event through the 45-day follow-up visit was significantly lower for patients discharged on warfarin alone (HR: 0.692; 95% CI: 0.569–0.841) and DOAC alone (HR: 0.731; 95% CI: 0.574–0.930) as compared with VKA and aspirin [37].

The limited available data supporting the use of OAC alone after LAAC does not allow for identification of which patients might benefit from this antithrombotic regimen. Dedicated RCTs aimed at testing different post-LAAC drug regimens including OAC only, are ongoing (Table 3).

**Table 3.** **Ongoing randomized clinical trials comparing different post-LAAC antithrombotic regimens**.

**Study name**	**Identific ation number**	**Study arms**	**Study population**	**Sam ple size**	**Primary outcome**	**Expect ed primary outcome achieved**
ANDES	NCT03568890	DAPT for 8 weeks vs. DOAC for 8 weeks	Patients eligible for short-term OAC submitted to successful LAAC*	350	DRT at 2-month TEE	09.2023
ADALA	NCT05632445	DAPT for 3 months vs. Apixaban for 3 months	Patients eligible for short-term OAC submitted to successful LAAC*	160	Composite of thromboembolic events, DRT and major bleeding events at 3 months after LAAC	Achieved
FADE-DRT	NCT04502017	half-dose DOAC vs. OAC for 6 weeks followed by standard DAPT until 6 months vs. OAC for 6 weeks followed by ASA+Clopidogrel (if Responders) or ASA+half-dose DOAC until 6 months	Patients eligible for short-term OAC submitted to successful LAAC*	360	Composite of Stroke, Systemic Embolism, and DRT at 1 year	12.2022
					Major bleedings at 1 year	
ASPIRIN-LAAO	NCT03821883	Aspirin vs. placebo (at 6 months after LAAC)	Patients submitted 6 months earlier to Watchman device implantation and without indication for long-term Aspirin	1120	Stroke, systemic embolism, CV/unknown death, acute coronary syndrome, coronary or periphery artery disease requiring revascularization, major bleeding at 2 years after randomization	06.2022

*Successful LAAC is defined as lack of relevant procedural complications. DAPT, dual antiplatelet therapy; DOAC, direct oral anticoagulant; OAC, oral anticoagulation; DRT, device related thrombus; TEE, transesophageal echocardiography; LAAC, left atrial appendage closure; ASA, aspirin.

### 2.3 Dual Antiplatelet Therapy

Dual antiplatelet therapy (DAPT) is a common antithrombotic drug regimen prescribed after LAAC in clinical practice, especially in Europe. Accordingly, this discharge treatment was the most used in the context of the two largest multicenter real-life LAAC studies conducted outside of the US [14, 15]. The Ewolution study was a multicentre, prospective, non-randomized cohort study including 1025 patients undergoing LAAC with the Watchman 2.5 device [14]. The study was conducted in a high risk AF population as witnessed by the population age (more than half of the patients were older than 75 years), the history of ischemic/hemorrhagic stroke (in more than one-third of the population), the mean CHA2DS2-VASc (4.5 ± 1.6) and HASBLED (2.3 ± 1.2) scores (significantly higher as compared to those of previous large multicenter studies conducted in US with the same device). The study showed promising results in terms of technical success (98.5%), procedural safety (2.7% of procedural complications) and annual stroke rate (1.4% vs. an expected rate based on CHA2DS2-VASc score of 7.5%). Unlike in the two initial Watchman RCTs and their subsequent continued access registries [9, 10, 40], the majority of patients were discharged under DAPT (60%) followed by OAC alone (27%), SAPT (7%) and no therapy (6%). Of note, no patients were discharged under combined OAC+SAPT therapy. The 2-month TEE showed DRT in 3.7% of patients without being correlated to the discharge antithrombotic regimen (*p* = 0.14). Consistently, no difference was observed between the different discharge drug regimens in terms of death, stroke or bleeding rates at 1 year after LAAC [41]. Of note, major bleeding was the most common adverse event observed at one year (2.5%), especially within the first 6 months, with a significant reduction in the subsequent months after switching to aspirin monotherapy. Similar results were observed in the other large prospective multicenter observational study performed outside of the US with the Amulet device [39]. The Amulet Observational Study included 1088 high risk AF patients (75 ± 8.5 years, 64.5% male, mean CHA2DS2-VASc: 4.2 ± 1.6, mean HAS-BLED: 3.3 ± 1.1) undergoing LAAC with implantation of Amulet device. As observed in the Ewolution study, the majority of patients were discharged under DAPT (54.3%) with very encouraging outcomes despite the high risk population treated and the low percentage of patients discharged under OAC (approximately one-tenth): 3.2% of procedural complications, 1.5% of DRT at 1–3 month-TEE (well distributed among the different discharge antithrombotic regimens) and ischemic stroke (2.2% observed vs. 6.7% expected based on CHA2DS2-VASc Score). Again, the amount of safety events reported at two years reflected the high risk population enrolled in the study: mortality at 2 years was 15.2% (with only one-third due to cardiovascular death) whereas the major bleeding annual rate was 7.2% after 2 years (significantly higher if compared to previous large registries most likely due to higher bleeding risk population: 72% with history of major bleeding). Of note, the major bleeding rate during the first year amounted to 10.1% with the majority of bleeding events occurring within 3 months after LAAC, in patients discharged under DAPT [39, 42].

Søndergaard *et al*. [30]. compared in a cohort of 1527 patients treated with Watchman, both safety and efficacy outcomes of the combined therapy OAC (95% VKA) plus Aspirin versus antiplatelet therapy (91% on DAPT) by using a propensity score matching analysis. At 6 months, there were no differences between groups in terms of non-procedural thromboembolic events (98.8% vs. 99.4%; *p* = 0.089) or major bleeding (95.7% vs. 95.5%; *p* = 0.775). However, DRT was higher in the antiplatelet group (3.1% vs. 1.4%; *p* = 0.014), even after excluding patients discharged under SAPT (3.3% vs. 1.1%, *p* = 0.005) [30].

The optimal duration of DAPT after LAAC still remains unknown. In the Amulet Observational Study almost half of patients discharged under DAPT switched to SAPT within 3 months after LAAC, mainly due to extreme bleeding risk or recurrent bleeding episodes [39].

Finally, short DAPT (1–3 months) followed by SAPT is a common antithrombotic regimen after LAAC outside of the US and supported by means of several large multicenter observational studies to be a valid alternative to the standard combined antithrombotic regimen in patients not eligible for short-term OAC. However, the duration and the target population of this regimen still need to be clarified.

### 2.4 Single Antiplatelet Therapy

In clinical practice, the majority of patients referred to LAAC are at high bleeding risk. Some patients may carry a risk of life-threatening or disabling bleeding due to the persistence of comorbidities/conditions associated with an extreme major bleeding risk including diffuse intracranial amyloid angiopathy, history of intracranial bleeding, special blood cell dyscrasia, bowel angiodysplasia, or a history of recurrent GI bleedings.

Nielsen-Kudsk *et al * [18]. identified from the Danish Stroke Registry 302 matched patients including 151 AF patients with a history of intracranial bleeding undergoing LAAC and 151 AF patients with a history of intracranial bleeding undergoing “standard therapy” (with only 20% of them under OAC and all the remaining patients under either SAPT or no antithrombotic therapy) without LAAC. The mean age (72 years) and risks for stroke (mean CHA2DS2-VASc score: 3.9) and bleeding (mean HAS-BLED score: 4.2) were similar in this matched cohort. Patients treated with LAAC (discharged under SAPT in 93% of cases) had a lower risk of the composite of all-cause mortality, ischaemic stroke and major bleeding as compared to patients treated with standard medical care (HR: 0.16; 95% CI: 0.07–0.37) [18].

The concern of prematurely switching from DAPT to SAPT or SAPT only therapy following LAAC is that it might increase the risk of DRT. However, the so far limited evidence does not support these concerns. In a monocentric experience including 107 consecutive patients treated with LAAC and discharged in most cases (88%) under SAPT, Korsholm *et al*. [19] observed a relatively low rate of DRT (1.9%), stroke (2.3%) and bleeding (6.5%) after a median follow-up of 2.3 years. Furthermore, Pouru *et al*. [20] showed in a monocentric study including 165 consecutive patients who underwent LAAC and discharged under SAPT; a low annual rate of major bleedings (3.6%) and cerebrovascular events (1.7%) after 3 years of follow-up. Finally, Patti *et al*. [21] showed in a retrospective multicenter observational study including 610 consecutive LAACs that SAPT as compared to DAPT was independently associated with reduction of major bleeding (2.9% vs. 6.7%, *p* = 0.038; adj HR 0.37; 95% CI: 0.16–0.88; *p* = 0.024), with no significant excess in the composite of major adverse cardiovascular events or DRT (7.8% vs. 7.4%; adj HR 1.34; 95% CI: 0.70–2.55; *p* = 0.38) and no difference in DRT, although the frequency of DRT appeared lower than expected (SAPT 0.7% vs. DAPT 0.9%, *p* = 0.38).

In conclusion, SAPT represents a regimen that might be considered at discharge in patients undergoing LAAC due to very high bleeding risk with the important caveat that RCTs in this population are lacking.

### 2.5 No Antithrombotic Therapy

Data related to patients discharged after LAAC without antithrombotic therapy are scarce. Only 6% and 2% of Ewolution and Amulet Observational Study populations respectively received no antiplatelet therapy and neither baseline characteristics nor clinical outcomes at follow-up of these small subgroups were reported. Certainly, this subgroup of patients were at prohibitive bleeding risk or experienced periprocedural major bleeding. In the latest European expert consensus document on LAAC, the complete abandonment of antiplatelet or anticoagulation therapy following LAAC is strongly discouraged with the suggestion of a minimal period of SAPT during 2–4 weeks or consideration of alternative LAAC approaches (either surgical or hybrid LAAC) [43].

## 3. Special Clinical Scenarios

DRT and residual PDL represent the most common LAAC device complications in clinical practice. Evidence related to their association with increased thromboembolic risk are accruing [44, 45, 46]. Management of post-LAAC drug regimen in these particular clinical scenarios will be discussed below.

### 3.1 Device Related Thrombus

The most feared LAAC device complication is DRT due to its associated thromboembolic risk [45, 46, 47]. DRT is defined as a homogeneous echo-dense mass adherent to the atrial surface of the LAAC device detected by TEE in multiple projections [48]. Recent studies showed that Cardiac computed tomography (CT) might be a potential alternative to TEE for the detection of DRT [49]. An analysis of the two pivotal RCTs and their subsequent continuous access registries including 1739 patients submitted to LAAC (7159 patient-years follow-up) and followed with serial TEEs (at 45 days, 6 and 12 months) showed an overall DRT incidence of 3.74% [47]. The timing of DRT detection varies among the different studies, most likely as a consequence of the different post-LAAC drug regimen and imaging protocol used: in the above analysis, Dukkipati *et al*. [47] observed almost one-third of the cases by means of unplanned TEE whereas half of the DRT detected in the context of planned TEE were observed after 6 months, therefore suggesting that DRT prevention should be considered even at long-term.

Several patient baseline (e.g., reduced ejection fraction, history of stroke) and LAAC procedural characteristics (e.g., device deep implantation), have emerged as consistent predictors of DRT and therefore need to be considered at the time of prescribing post-procedural antithrombotic therapy [46, 47]. Although the studies testing so far the impact of post-LAAC regimen on DRT occurrence have shown controversial results [30, 46, 50], it seems that short-term OAC usage after LAAC might reduce DRT incidence [30].

The management of DRT is challenging. Both Protect AF and Prevail trials mandated the use of VKA to treat DRT. However, the majority of patients currently submitted to LAAC are not eligible even for short-term OAC. As a consequence, large multicenter cohort studies including DRT cases report heterogeneous strategies to manage DRTs. Sedaghat *et al*. [45] showed in the multinational EUROC-DRT registry including 156 patients with DRT, that the majority of patients were treated by OAC (32.1% with DOAC and 22.3% with VKA) followed by heparin (31.3%), antiplatelet therapy (6.3%) and no antithrombotic therapy (1.8%). A complete DRT resolution was achieved in almost 80% at approximately 3 months after DRT detection with comparable resolution rates between the different initial treatment regimens (SAPT: 57.1%, DAPT: 85.7%, VKA: 80.0%, DOACs: 75.0%, Heparin: 68.6%). Of note, the incidence of stroke and mortality at 1 year after LAAC was significantly higher in patients without complete DRT resolution (stroke: 17.6% vs. 6.5%, *p* = 0.09; mortality: 15.0% vs. 1.4%, *p* = 0.01). Bleedings under DRT treatment occurred in almost one-tenth of patients (9.8%) with the majority of them occurring under DOAC (54.5%) or heparin (36.4%) therapy [45]. A recent large retrospective multicenter cohort study including 237 DRTs and 474 controls observed a similar percentage of DRT resolution as compared to the above study (74.6% vs. 80%) [46]. However, unlike what was observed by Sedaghat *et al*. [45], DRT resolution did not improve prognosis [46].

Whether DRT is directly causative for adverse events remains speculative. However, as suggested by the IFU of Watchman 2.5/FLX, restart/continue OAC until DRT resolution should always be considered in the absence of an excessive bleeding risk.

### 3.2 Residual Peridevice Leak

Residual PDL is the most common device-related finding after LAAC and consists of a gap at the LAAC device sides allowing residual communication between LAA and circulation. It can be detected by using TEE or computed tomography [38]. The reported incidence significantly varies among the different multicenter studies (1.8–54%) [9, 10, 11, 15, 38] for several reasons, including the imaging method/timing and the PDL definition used, the central assessment, the study design and the device implanted. Unlike DRT, the clinical relevance of PDL is a matter of ongoing debate [44, 51]. Study protocols of the two pivotal Watchman trials recommended continuation of OAC even later than 45 days after LAAC in the presence of PDL >5 mm at TEE follow-up. This PDL size was arbitrarily chosen as a reasonable cut-off value although the long-term clinical consequences of such flow were unknown. A post-hoc analysis of Protect-AF trial including 445 patients successfully treated with Watchman implantation and performing 45-day TEE, showed that a composite of stroke, systemic embolism, or cardiovascular or unexplained death was not significantly different between patients with versus without residual PDL (2.0% vs. 2.8%; *p* = 0.635). Furthermore, no statistical interaction between the severity of PDL and the composite endpoint was observed [51]. Although the results suggested that stopping OAC at 45 days regardless of the PDL presence might be safe, the authors recommended taking these findings with caution due to the small statistical power and the potential bias generated by the OAC continuation in some patients with residual PDL. In the much larger cohort (n = 51,333) derived from National Cardiovascular Data Registry LAAO Registry, Alkhouli *et al*. [44] compared patients with vs. without PDL at 45-day TEE in terms of thromboembolic events. Compared with patients with no leak, those with small leaks (0–5 mm) had slightly higher odds of thromboembolic events (adj HR: 1.152; 95% CI: 1.025–1.294). Of note, large leaks (>5 mm) were not associated with an increased risk of adverse events, although higher proportions of these patients were maintained under OAC [44].

Based on the above study it appears that OAC might mitigate the increased thromboembolic risk associated with residual PDL. However, the majority of patients undergoing LAAC are at high bleeding risk and eligibility for OAC is limited. A feasible alternative in this scenario is the percutaneous closure of the residual shunt. Short-term outcomes of this intervention were recently reported in several multicenter studies [52]. Piayda *et al*. [52] included 95 patients with residual PDL and percutaneous closure and reported a technical success of 100% with no major complications. At follow-up, persistent leaks were found in 18.9% of patients, although PDLs were significantly reduced in size with no leak >5 mm [52].

No dedicated study has specifically assessed the net clinical benefit of continuing OAC or performing percutaneous PDL closure in patients with residual PDL after LAAC. Recommendations related to the management of antithrombotic therapy in this particular scenario are therefore based on expert opinion. In the latest consensus document, it was left at the discretion of operators to decide between restarting OAC versus percutaneous closure of relevant PDL (≥5 mm) [53]. In our view, the best approach for PDL management remains its prevention which might be improved by devices and procedure iterations [54] by optimizing the procedural planning [55] or the procedural guidance [56].

## 4. Future Directions

**Fig. 1.**
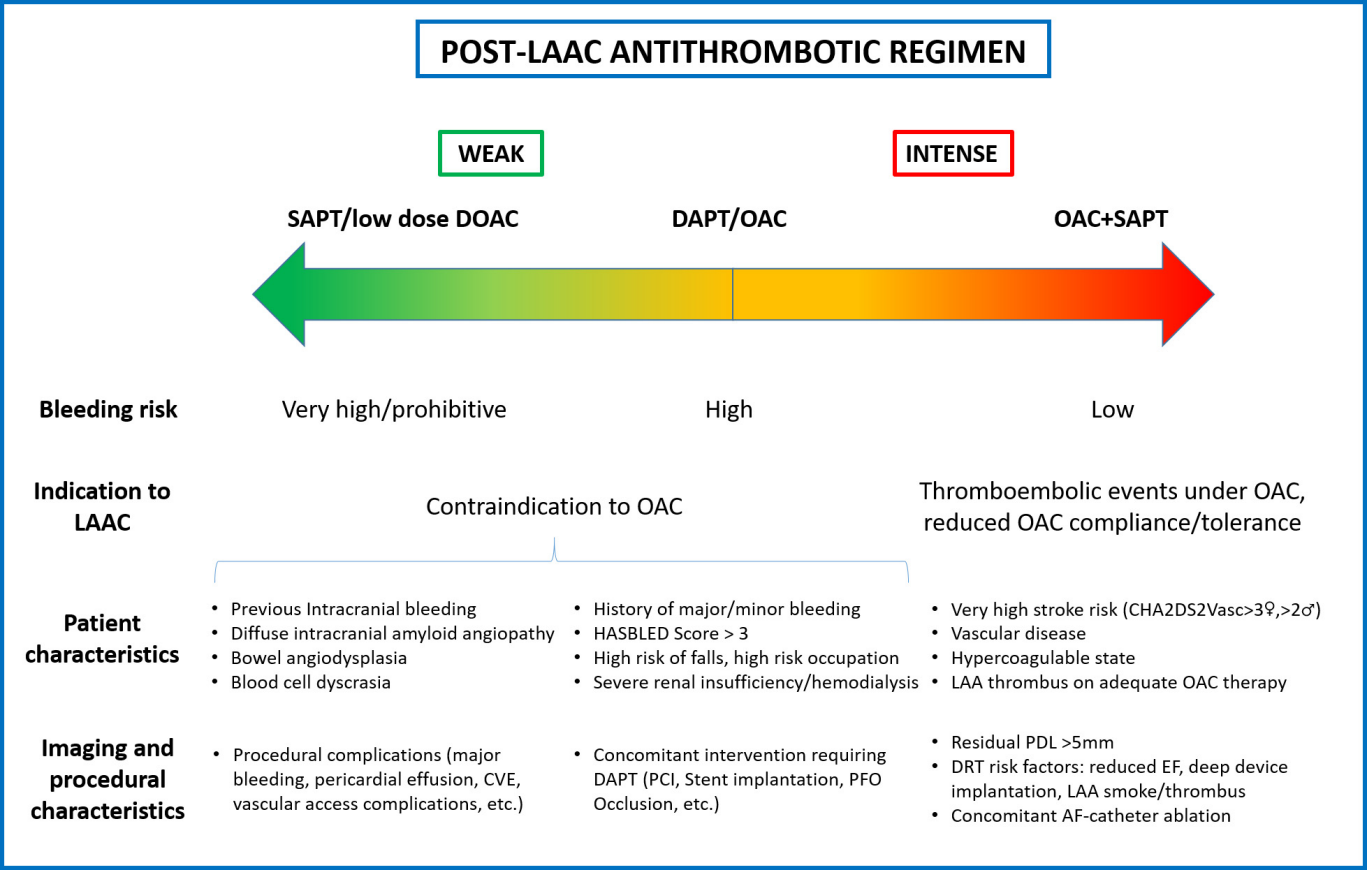
**The evidence so far available suggests that antithrombotic therapy after LAAC should be tailored based on several parameters, including patient characteristics and procedural outcomes**. LAAC, left atrial appendage closure; SAPT, single antiplatelet therapy; DOAC, direct oral anticoagulation; DAPT, dual antiplatelet therapy; OAC, oral anticoagulant; LAA, left atrial appendage; CVE, cerebrovascular events; PCI, percutaneous coronary intervention; PFO, patent foramen ovale; PDL, peri-device leak; DRT, device related thrombus; EF, ejection fraction; AF, atrial fibrillation.

A standard post-LAAC drug regimen able to match the large heterogeneity of patients undergoing LAAC population appears unlikely. The evidence so far available and the assumed multifactorial DRT underlying pathophysiology, suggest that antithrombotic therapy after LAAC should be tailored based on the ischemic and bleeding risk and procedural outcomes (Fig. 1). Several RCTs adequately powered for clinical outcomes are currently ongoing to broaden our knowledge on this topic (Table 3).

The ANDES Study (clinicaltrial.gov. NCT03568890) is an ongoing RCT including 350 patients deemed eligible for short-term OAC and submitted to a successful LAAC comparing 8 weeks DOAC with 8 weeks DAPT in terms of DRT as evaluated by 45-day TEE. In a similar population but of smaller size (n = 160), the ADALA Study (clinicaltrial.gov. NCT05632445) will compare 3 months DAPT with 3 months Apixaban in terms of composite of thromboembolic events, DRT and major bleeding events at 3 months after LAAC. FADE-DRT Study (clinicaltrial.gov. NCT04502017) is a multicenter RCT comparing three different post-LAAC regimens, including half-dose OAC vs. 6 weeks OAC followed by 4.5 months of standard DAPT vs. 6 weeks OAC followed by 4.5 months of DAPT guided by a genetic test, in terms of 2 primary endpoints: composite of stroke, systemic embolism and DRT at 1 year or major bleedings at 1 year after LAAC. The population included will be similar to the above two trials, i.e., consisting of patients eligible for short-term OAC. The ASPIRIN-LAAO trial (clinicaltrial.gov NCT03821883) is a multicenter RCT double-blinded, placebo-controlled study that investigates the effects (in terms of both ischemic and bleeding events at 2 years after randomization) of stopping aspirin six months after LAAC. In this study all bleeding risk category patients will be included, and participants will be randomized 6 months after successful implantation of the Watchman device to receive Aspirin or Placebo.

In the coming years, new antithrombotic drugs might be considered after LAAC to prevent the occurrence of DRT and mitigate the antithrombotic drugs bleeding risk associated. Factor XI inhibitors are emerging as a new attractive antithrombotic strategy in AF patients with the potential to uncouple the pharmacological effect and the adverse events of anticoagulant therapy. The rationale supporting these new drugs is related to their differential contribution to thrombus amplification (in which it plays a major role) and hemostasis (where these drugs are only marginally involved). PACIFIC-AF was a phase 2 dose-finding multicenter RCT comparing 2 oral doses of asundexian (20 or 50 mg) with apixaban in 755 patients with AF, increased CHA2DS2-VASc score and at least one bleeding risk, including history of previous bleeding requiring medical attention within 12 months, estimated glomerular filtration rate of 30–50 mL/min, or current indication for aspirin. The study showed at 4 weeks a significant reduction in terms of relevant bleeding events in the pooled asundexian versus apixaban groups (0.33 [0.09–0.97]) [57]. However, no studies are currently ongoing to test the utility of these new drugs in preventing DRT after LAAC.

Finally, the hemostatic abnormalities in DRT patients should be better investigated to potentially facilitate the personalization of antithrombotic therapy and improve net clinical outcomes [58].

## 5. Conclusions

Percutaneous LAAC requires antithrombotic therapy after device implantation to prevent DRT while endothelialization occurs. The variety of post-procedural antithrombotic regimens currently used is high and includes OAC with single antiplatelet therapy or dual antiplatelet therapy. The majority of patients treated with LAAC, especially outside of the US, carry a high bleeding risk and do not tolerate the standard treatment suggested in the instructions for use, i.e., anticoagulant plus aspirin for 45 days or DAPT for 90 days. Robust data on the optimal post-procedural antithrombotic regimen in patients is sparse, making clinical decisions challenging. The observational evidence so far available suggests that antithrombotic therapy after LAAC should be adapted according to the bleeding and ischemic risk and the procedural result. Short DAPT or even SAPT may be safe in high bleeding risk patients, whereas a continued OAC may be the treatment of choice in patients with low bleeding risk but high risk for recurrent stroke. RCTs comparing different post-LAAC drug regimens in high bleeding risk patients with adequate power for clinical and imaging endpoints (i.e., DRT), represents the most needed gap to be closed in the near future.
